# Sulfonamide Inhibition Studies of the β-Class Carbonic Anhydrase CAS3 from the Filamentous Ascomycete *Sordaria macrospora*

**DOI:** 10.3390/molecules25051036

**Published:** 2020-02-25

**Authors:** Daniela Vullo, Ronny Lehneck, William A. Donald, Stefanie Pöggeler, Claudiu T. Supuran

**Affiliations:** 1 Dipartimento di Chimica Ugo Schiff, Università degli Studi di Firenze, 50019 Sesto Fiorentino (Florence), Italy; daniela.vullo@unifi.it; 2 Institute of Microbiology and Genetics, Department of Genetics of Eukaryotic Microorganisms, Georg-August-University, 37077 Gottingen, Germany; ronnylehneck@hotmail.de (R.L.); spoegge@gwdg.de (S.P.); 3 University of New South Wales, School of Chemistry, Sydney, NSW 2052, Australia; w.donald@unsw.edu.au; 4 Neurofarba Dept., Section of Pharmaceutical and Nutriceutical Sciences, Università degli Studi di Firenze, 50019 Sesto Fiorentino (Florence), Italy

**Keywords:** carbonic anhydrase, fungus, sulfonamide, kinetics, antifungals, *Sordaria macrospora*

## Abstract

A new β-class carbonic anhydrase was cloned and purified from the filamentous ascomycete *Sordaria macrospora,* CAS3. This enzyme has a higher catalytic activity compared to the other two such enzymes from this fungus, CAS1 and CAS2, which were reported earlier, with the following kinetic parameters: k_cat_ of (7.9 ± 0.2) × 10^5^ s^−1^, and k_cat_/K_m_ of (9.5 ± 0.12) × 10^7^ M^−1^∙s^−1^. An inhibition study with a panel of sulfonamides and one sulfamate was also performed. The most effective CAS3 inhibitors were benzolamide, brinzolamide, dichlorophnamide, methazolamide, acetazolamide, ethoxzolamide, sulfanilamide, methanilamide, and benzene-1,3-disulfonamide, with K_I_s in the range of 54–95 nM. CAS3 generally shows a higher affinity for this class of inhibitors compared to CAS1 and CAS2. As *S. macrospora* is a model organism for the study of fruiting body development in fungi, these data may be useful for developing antifungal compounds based on CA inhibition.

## 1. Introduction

The filamentous ascomycete *Sordaria macrospora* is a coprophilous fungus that naturally lives on herbivore dung. For many years *S. macrospora* served as a model organism to study fruiting body development in fungi [1]. Previous studies identified four carbonic anhydrase (CA, EC 4.2.1.1) genes in the genome of *S. macrospora* that are designated as *cas1*, *cas2*, *cas3*, and *cas4* [2,3,4,5]. The two β-CA genes *cas1* and *cas2* have high sequence identity and encode enzymes with characteristics of the plant-like sub-class of β‑CAs [2]. The β-CA *cas3* belongs to the cab-like [6] sub‑class, whereas *cas4* encodes for an α-class CA. CAS1 and CAS3 are cytoplasmic enzymes, while CAS2 is located in the mitochondria, and CAS4 is a secreted protein [3,4,5]. The three β-CAs *cas1*, *cas2*, and *cas3* are involved in the sexual development of *S. macrospora* [3]. Deletion of the α-CA *cas4* resulted in a significantly reduced rate of ascospore germination but showed no significant involvement in sexual development and vegetative growth [5]. Thus, the detailed physiological roles of all these enzymes are not yet entirely elucidated.

The CA metalloenzymes are ubiquitous in most life forms, with eight distinct genetic families encoding the α-, β-, γ-, δ-, ζ-, η-, θ-, and ι-CAs [7,8,9,10,11,12,13]. By catalyzing the hydration of CO_2_ to bicarbonate and protons, CAs are involved in various processes, starting with pH regulation and ending with metabolism [4,7,8,9,10,11,12,13]. In fungi, they play crucial roles in growth, development, virulence, and survival [10,11], and modulation of their activity with inhibitors and/or activators was proposed as a new approach for designing antifungals [10,11,14,15,16]. The metal ion from the enzyme active site is usually coordinated by three amino-acid residues, whereas the fourth ligand is a water molecule or hydroxide ion which acts as a nucleophile in the hydrolytic reactions [4,7,8,9,10,11,12,13]. In β-CAs the Zn(II) is coordinated by two Cys residues, one His residue, and one water molecule/hydroxide ion [17,18].

The two β-class CA proteins CAS1 and CAS2 representing the major CA proteins of *S. macrospora* were biochemically and structurally characterized by our groups [3,17,18]. Both proteins could be easily produced in *Escherichia coli* and obtained in high purity (5–10 mg of CAS1 and 10–20 mg of CAS2 per L of culture) and exhibited noticeable in vitro CO_2_ hydration activity with *k*_cat_/*K*_m_ for CAS1 of 1.30 × 10^6^ M^−1^∙s^−1^ and for CAS2 of 1.21 × 10^6^ M^−1^∙s^−1^ [17]. The crystal structures of CAS1 and CAS2 were determined to a resolution of 2.7 Å and 1.8 Å, respectively [17]. The oligomeric state of both proteins was tetrameric. With exception of the active site composition, no further major differences could be observed between the structures of CAS1 and CAS2. The activity of both CAs was only weakly inhibited by nitrite and nitrate anions and some other anions, making them good candidates for industrial applications [17]. In addition, both enzymes were recently investigated for their inhibition with a panel of 39 aromatic, heterocyclic, and aliphatic sulfonamides and one sulfamate, which acted in some cases as rather efficient inhibitors [18]. Dorzolamide, brinzolamife, and acetazolamide (**AAZ**) were medium-potency, high-micromolar inhibitors of these enzymes, being among the most effective ones detected so far [18]. However, no studies on the purification, catalytic activity, and inhibition of CAS3 are available to date. Here, we describe the purification, kinetic characterization, and inhibition studies of the cab-like β-CA of *S. macrospora* CAS3, with a panel of sulfonamides/sulfamates.

## 2. Results and Discussion

**Figure 1:**
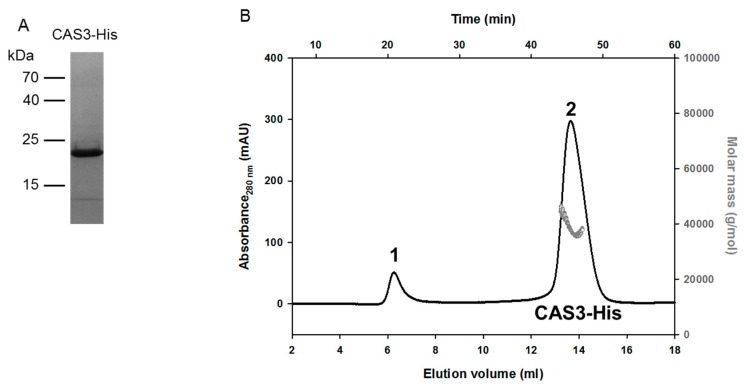
Purification and size-exclusion chromatography (SEC) of carbonic anhydrase 3 (CAS3)-His on a Superdex 200 10/300 column coupled with multi-angle laser light scattering. (**A**) Coomassie-stained, 15% SDS gel of purified CAS3-His. After washing of unbound proteins, the His-tagged enzymes were eluted by addition of 500 µL of elution buffer. Then, 10 µL of the protein solution was separated by SDS-PAGE. The expected protein size is 20.36 kDa. (**B**) Size-exclusion chromatography CAS3-His (black line) on a Superdex 200 10/300 column coupled with multi-angle laser light scattering (gray line). The reference proteins for SEC had the following elution profile: 670 kDa = 8.16 mL (thyroglobulin), 158 kDa = 12.54 mL (γ-globulin), 44 kDa = 15.08 mL (ovalbumin), 17 kDa = 17.21 mL (myoglobin), 1.35 kDa = 20.46 mL (vitamin B12). The calculated molar mass of CAS3 amounts to 38,650 g∙mol^−1^ (±0.1%), which corresponds to 1.9 times of the CAS3 monomer (20.36 kDa) (C). Peak 1 in the CAS3 elution profile is very likely due to protein aggregates that eluted close to the void volume of the column. Peak 2 represents the CAS3-His protein.

The protein encoded by the *cas3* gene belongs to the cab-like sub-class of β-CAs and is localized to the cytoplasm [3]. Indeed, the cab-like CAs take their name from the enzyme discovered in the archaeon *Methanobacterium thermoautotrophicum*, which has only one such enzyme, called cab [9], and they are amongst the smallest CAs ever reported. Similar to cab, CAS3 that is reported here is composed of 174 amino-acid residues, with a calculated molecular mass of 19.2 kDa. CAS3 was synthesized in *E. coli* Rosetta (DE3) cells as a C-terminal His-tag fusion protein (Figure 1). After purification, 5–7.5 mg CAS3 could be obtained per L of culture. The purified enzyme was dialyzed against 50 mM 4-(2-hydroxyethyl)-1-piperazineethanesulfonic acid (HEPES) pH 8.3, 50 mM NaCl. To analyze the state of CAS3-His in solution, size‑exclusion chromatography and multi-angle laser light scattering (SEC-MALLS) was performed (Figure 1). The calculated molar mass of CAS3 amounts to 38,650 g∙mol^−1^ (±0.1%), which corresponds to 1.9 times of the CAS3 monomer (20.36 kDa) (Figure 1). These findings suggest that the biological unit of CAS3 is a homo-dimer in solution, which is the same as for cab and other β-CAs from fungi or bacteria that have been investigated to date [19,20,21,22,23].

The catalytic activity for the physiological CO_2_ hydration reaction that is catalyzed by these enzymes was investigated for the purified CAS3 by using a stopped-flow assay [24]. The activity of CAS3 was compared to those of the other two β-CAs present in the genome of this organism (CAS1 and 2), as well as with other similar enzymes from fungi/yeasts (Can2 from *Cryptococcus neoformans* [22], CalCA from *Candida albicans* [25], SceCA from *Saccharomyces cerevisiae* [26]), as well as the widespread human isoforms hCA I and II, belonging to the α-class [7]. As seen from data of Table 1, CAS3 has a catalytic activity that is an order of magnitude higher than CAS1 and CAS2, with the following kinetic parameters: k_cat_ of (7.9 ± 0.2) × 10^5^ s^−1^, and k_cat_/K_m_ of (9.5 ± 0.12) × 10^7^ M^−1^∙s^−1^. The activity of CAS3 is, thus, similar to that of CalCA and SceCA, being almost two times higher than that of the “slow” human isoform hCA I, a highly abundant enzyme in red blood cells [27]. Only hCA II among the investigated enzymes shown in Table 1 has a higher catalytic activity, but this enzyme is known to be one of the most effective catalysts in nature [7,8,9]. In addition, the CO_2_ hydrase activity was effectively inhibited by the clinically used sulfonamide acetazolamide, one of the most investigated CA inhibitors (CAIs) to date [7,8] (Table 1). Thus, apparently, CAS3 also has a higher affinity for sulfonamide inhibitors compared to CAS1 and 2, for which acetazolamide was a rather weak inhibitor, with K_I_s in the range of 445–816 nM (Table 1) [17,18].

**Figure 2:**
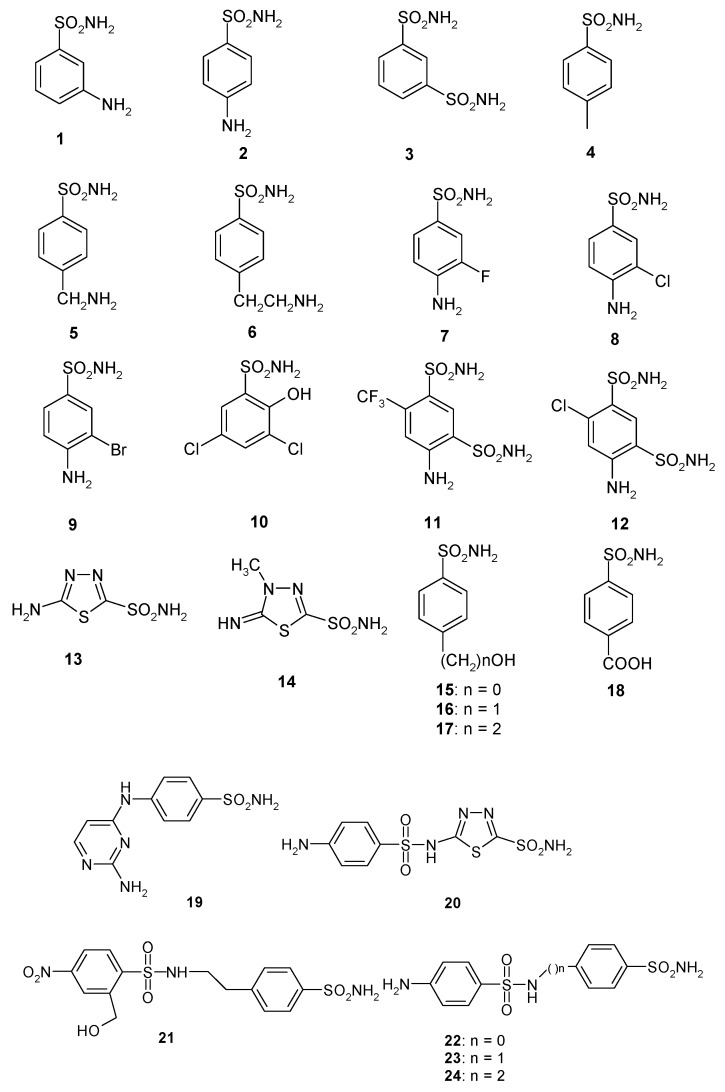

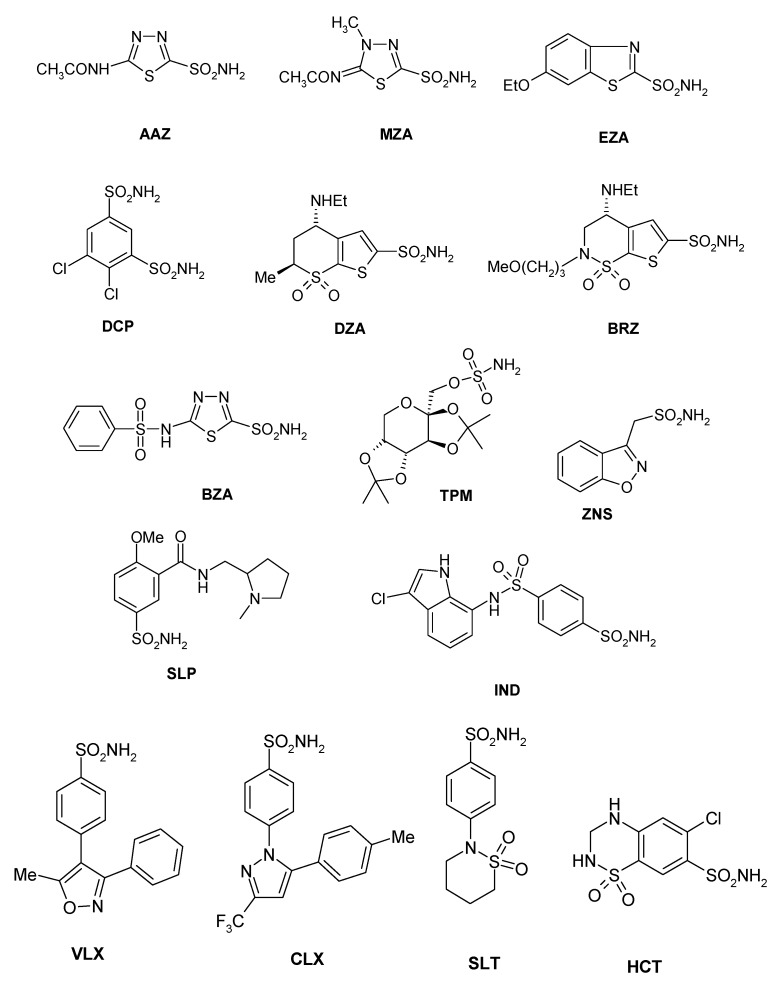
Structure of sulfonamide and sulfamate inhibitors of types **1–24** and **AAZ–HCT** investigated in the present study.

We, thus, conducted a detailed inhibition study of CAS3 with a panel of sulfonamides and one sulfamate, of types **1–24** and **AAZ–HCT** (Figure 2). The latter compounds are in fact clinically used drugs as diuretics, antiglaucoma, antiepileptic, and antiobesity agents. They target some of the many human isoforms involved in these pathologies [28,29,30,31,32,33,34].

As seen from data of Table 2, sulfonamides/sulfamates **1–24** and **AAZ–HCT** inhibit CAS3 with various efficacies. Generally, they are active in the high nanomolar range, except sulthiame **SLT** and **8** which were micromolar inhibitors (K_I_s in the range of 2.07–4.83 µM). Many of the investigated compounds, both among the simple derivatives **1–18** or the more complex structures **19–24** or the clinically used agents, inhibited CAS3 with K_I_s < 100 nM, typically in the range of 54–95 nM. To this category of effective inhibitors belong the following derivatives: **1–3** (simple amino-benzenesulfonamides and benzene-1,3-disulfonamide); **12–14** (again, a benzene-1,3-disulfonamide derivative, **12**, and the deacetylated precursors of acetazolamide and methazolamide, **13** and **14**); **16** (4-hydroxymethyl-benzenesulfonamide); **19–24** (an amino-pyrimidinyl-sulfanilamide, **19,** and the sulfanilyl-substituted aromatic/heterocyclic sulfonamides **20–24**, all of them possessing a quite elongated moiety); **AAZ**, **MZA**, **EZA**, **DCP**, **BRZ**, **BZA,** and **IND** (clinically used or preclinical (**IND**) CAIs, possessing simple or more elaborated scaffolds). Thus, the structure–activity relationship (SAR) for CAS3 inhibition is not simple, since a rather large number of structurally different compounds show this profile of effective inhibitors. However, it may be observed that, with few exceptions, CAS3 is the isoform with the highest affinity for sulfonamides among the three *S. macrospora* isoforms investigated to date. Only CAS2 in some cases is more effectively inhibited by some compounds (compared to CAS3) [17,18], for example, with **13, 15, 17,** and **SLT** (Table 2).

The remaining compounds showed less effective inhibition of CAS3, with K_I_s ranging between 191 and 831 nM. In this category of weak–medium inhibitors were the following compounds: **4–7; 9–11; 15; 17; 18; DZA; TPM–SLP; VLX; CLX; SAC; HCT**. Again, they belong to a large variety of structural classes, from simple aromatic derivatives (**4–7, 15, 17, 18**) to heterocyclic ones, incorporating mono- or bicyclic ring systems (**DZA, ZNS, VLX, CLX**) to a sugar sulfamate (**TPM**), and the SAR is not straightforward.

## 3. Materials and Methods

### 3.1. Construction of the cas3 Overexpression Vector

RNA from *S. macrospora* was isolated as described in Reference [3]. To remove obsolete genomic DNA, the RNA was treated with DNase I (Thermo Scientific, EN0521, Waltham, MA, USA) according to the manufacturer’s manual. The “Transcriptor High Fidelity cDNA Synthesis kit” (Roche, Basil, Switzerland) was used for the reverse transcription reaction. Template concentration was 2 µg of RNA. The complementary DNA (cDNA) of the *cas3* open reading frame (ORF; 525 bp) was amplified with primer pair CAS-pet-f (GGAGATATACATAATGATGCCCGTCACCAACGAAGA) and Cas3-pet-r (GGTGGTGGTGCTCGAGAACAACCCTCACCGTCTTGC). The obtained PCR fragment was cloned into the pET22b(+) expression plasmid (Novagen, Madison, WI, USA) linearized with *Nde*I and *Xho*I to create a 6×His fusion protein. The plasmid was named pET-CAS3.

### 3.2. Heterologous Expression of the cas3 Gene in E. coli

The production of the CAS3 protein was performed in *E. coli* strain Rosetta (DE3) (Invitrogen, Carlsbad, Germany). An overnight pre-culture was used to inoculate 4 × 0.5 L of Luria broth (LB) medium supplemented with 100 mg/L ampicillin and 0.5 mM ZnSO_4_. The cultures were grown to an OD_600_ of 0.1. Heterologous gene expression was then induced by the addition of 1 mM IPTG during the exponential phase of growth and lasted for 3–4 h at 30 °C. Subsequently, the cells were harvested (4000× *g*, 30 min, 4 °C) and flash‑frozen with liquid nitrogen and stored at −20 °C.

For determination of the protein concentration, 10 µL of the protein was mixed with 990 µL of Bradford reagent [35] and incubated for 2 min at RT. Then, the absorption was determined at 595 nm in a “Libra S12” (biochrom, Cambridge, UK) spectrophotometer in a 1-mL cuvette. Prior to this, a calibration line with bovine serum albumin was recorded.

The completeness of CAS3 was analyzed by mass spectrometry using the “LCQ DecaXP mass spectrometer” (Thermo Scientific, Waltham, MA, USA) and by SDS polyacrylamide gel electrophoresis (PAGE) [36]. CAS3 was separated on a 15% SDS PAGE with the protein standard (Pageruler™ Prestained Protein Ladder, SM0671, Thermo Fisher, Waltham, MA, USA). For visualization of the proteins after the electrophoresis, the gel was incubated for 30 min in SDS-gel staining solution (0.02% (*w*/*v*) Coomassie brilliant blue R250, 0.02% (*w*/*v*) Coomassie brilliant blue G250, 42.5% (*v*/*v*) ethanol, 0.5% (*v*/*v*) methanol, 10% (*v*/*v*) acetic acid), and afterward in SDS-gel distaining solution (45% (*v*/*v*) ethanol, 10% (*v*/*v*) acetic acid) for 30 min.

### 3.3. Purification of CAS3-His

For purification of CAS3, 10 g of *E. coli* cells were re-suspended in 30 mL of lysis buffer (20 mM imidazole, 50 mM NaH_2_PO_4_ pH 8, 300 mM NaCl, 0.02 mM MgCl_2_, one “Protease Inhibitor Cocktail” Tablet (Roche, Basil, Switzerland) and subsequently incubated for 30 min at 4 °C with a spatula tip of lysozyme (Serva, Heidelberg, Germany, 28262.03) and DNase I. After incubation, the cells were disrupted using a microfluidizer 110S (Microfluids, bonn, Germany). After centrifugation (50,000× *g*, 30 min, 4 °C), the clarified lysate was applied on an Ni-NTA agarose (Qiagen, Hilden, Germany, 1018244) column equilibrated with lysis buffer. Unbound proteins were removed by washing gradually with three column volumes (CV) of lysis buffer containing 40 mM, 60mM, 80 mM, and 100 mM imidazole. Bound CAS3 was eluted with lysis buffer containing 250 mM imidazole. Elution fractions were analyzed by SDS-PAGE, pooled, concentrated in a “Spin-X^®^ UF 20” (Corning, Kaiserslautern, Germany), and stored at 4 °C. After purification, 5–7.5 mg of CAS3 could be obtained per L of culture.

### 3.4. Size-Exclusion Chromatography Coupled with Multi-Angle Laser Light Scattering

Four hundred microliters CAS3 (3 mg/mL) were loaded separately on a 24-mL analytical “Superdex 200 (10/300)” gel filtration column using an “Äkta purifier” coupled to a “miniDAWN MALS” detector (Wyatt Technology, Santa Barbara, CA, USA). The column was pre-equilibrated with 50 mM NaH_2_PO_4_ pH 8.0, 250 mM imidazole, 300 mM NaCl. Multi-angle laser light-scattering analysis was performed continuously on the column eluate at 291 K (size-exclusion chromatography coupled with multi-angle laser light scattering, SEC-MALLS). Data analysis was carried out with Astra software (Wyatt Technology, Santa Barbara, CA, USA).

### 3.5. CA Inhibition Assay

An Applied Photophysics stopped-flow instrument was used for assaying the CA catalyzed CO_2_ hydration activity [24]. The pH indicator used was bromothymol blue at a concentration of 0.2 mM, working at the absorbance maximum of 557 nm. The buffer used was 20 mM Tris (pH 8.3), and 20 mM Na_2_SO_4_ was added to this buffer for maintaining constant the ionic strength (sulfate is not inhibitory and has a K_I_ > 200 mM against this enzyme; data not shown). The initial rates of the CA-catalyzed CO_2_ hydration were followed for a period of 10–100 s for each assay. The CO_2_ concentrations ranged from 1.7 to 17 mM for determining kinetic parameters of the reaction and the inhibition constants of tested inhibitors. For each measurement, six traces of the initial 5%–10% of the reaction were used for determining the initial velocity. Then, 10-fold decreasing dilution of inhibitor solutions, ranging from 1 nM and 100 µM were employed for determining the inhibition constants. Uncatalyzed rates were determined in the same manner and subtracted from total observed rates. Stock solutions of inhibitor (0.1 mM) were prepared in distilled deionized water, and dilutions up to 1 nM were done thereafter with the assay buffer. Inhibitor and enzyme solutions were preincubated together for 15 min at room temperature before to assay for allowing for the formation of E–I complexes. The inhibition constants were obtained by non-linear least-squares methods using the Cheng–Prusoff equation, and they represent the mean from at least three different determinations. The human isoforms hCA I, II were assayed in the same conditions as above except that the working pH was 7.4 HEPES buffer with phenol red as an indicator [21,22]. The inhibition constants measured by this method are in excellent agreement with the dissociation constants measured by native mass spectrometry [37,38].

## 4. Conclusions

Fungal CAs are of great interest both for biotechnological and pharmaceutical applications [10] because some of these enzymes are stable, relatively easy to produce, and may be used as model enzymes for testing inhibitors/activators. The organism *Sordaria macrospora,* used as a genetic model to study fruiting body development of filamentous fungi, encodes for at least four different CAs, two of which were thoroughly investigated earlier, CAS1 and CAS2. Here, we prove that CAS3, another representative enzyme from this organism, belonging to the β-CA class, may be of interest for better understanding the roles these proteins play in various physiologic processes of fungi. Unlike CAS1 and CAS2, which showed rather low catalytic activity for the hydration of CO_2_ to bicarbonate and protons, CAS3 is a highly effective catalyst, showing kinetic parameters comparable to those of other fungal/mammalian enzymes, i.e., k_cat_ of (7.9 ± 0.2) × 10^5^ s^−1^, and k_cat_/K_m_ of (9.5 ± 0.12) × 10^7^ M^−1^∙s^−1^. A detailed inhibition study of CAS3 with sulfonamides and one sulfamate allowed us to demonstrate that this enzyme has higher affinity for these inhibitors compared to CAS1 and CAS2, with clinically used agents such as benzolamide, brinzolamide, dichlorophnamide, methazolamide, acetazolamide, ethoxzolamide, sulfamilamide, metanilamide, and benzene-1,3-disulfonamide possessing K_I_s under 100 nM.

## Figures and Tables

**Table 1:** Kinetic parameters for the CO_2_ hydration reaction [24] catalyzed by the human cytosolic enzymes hCA I and II (α-class CAs) at 20 °C and pH 7.5, and the β-CAs Can2, CalCA (from *Cryptococcus neoformans* and *Candida albicans*, respectively), SceCA (from *Saccharomyces cerevisiae*), as well as the enzymes from *Sordaria macrospora*, CAS1–CAS3, measured at 20 °C, pH 8.3. Inhibition data with the clinically used sulfonamide acetazolamide (5-acetamido-1,3,4-thiadiazole-2-sulfonamide) are also provided.

Isozyme	Activity Level	K_cat_ (s^−1^)	K_cat_/K_m_ (M^−1^∙s^−1^)	K_I_ (Acetazolamide) (nM)
hCA I ^a^	Moderate	2.0 × 10^5^	5.0 × 10^7^	250
hCA II ^a^	Very high	1.4 × 10^6^	1.5 × 10^8^	12
Can2 ^b^	Moderate	3.9 × 10^5^	4.3 × 10^7^	10.5
CalCA ^c^	High	8.0 × 10^5^	9.7 × 10^7^	132
SceCA ^c^	High	9.4 × 10^5^	9.8 × 10^7^	82
CAS1 ^d^	Low	1.2 × 10^4^	1.30 × 10^6^	445
CAS2 ^d^	Low	1.3 × 10^4^	1.21 × 10^6^	816
CAS3 ^e^	High	(7.9 ± 0.2) × 10^5^	(9.5 ± 0.12) × 10^7^	94 ± 3

^a^ From References [7,8]; ^b^ from reference [22]; ^c^ from reference [12]; ^d^ from reference [17]; ^e^ this work: mean ± standard error, from three different assays.

**Table 2:** Inhibition of human isoforms hCA I and hCA II, and of the β-class fungal enzymes CAS1–CAS3 with sulfonamides **1–24** and the clinically used drugs **AAZ–HCT**, by a stopped flow CO_2_ hydrase assay [24].

Inhibitor/ Enzyme Class	K_I_* (nM)				
	hCA I^a^	hCA II ^a^	CAS1^b^	CAS2 ^b^	CAS3 ^c^
	α	α	β	β	β
**1**	28,000	300	361	386	90 ± 7.1
**2**	25,000	240	144	3480	84 ± 5.6
**3**	79 ^c^	8	225	3630	83 ± 6.0
**4**	78,500	320	47.1	6900	560 ± 21
**5**	25,000	170	323	8720	726 ± 30
**6**	21,000	160	241	7650	441 ± 14
**7**	8300	60	43.2	7360	585 ± 23
**8**	9800	110	79.6	9120	2078 ± 61
**9**	6500	40	580	12,000	712 ± 28
**10**	7300	54	>50,000	23,500	350 ± 13
**11**	5800	63	890	18,700	235 ± 16
**12**	8400	75	3350	>50,000	90 ± 5.7
**13**	8600	60	8650	48.1	88 ± 2.9
**14**	9300	19	7215	280	94 ± 4.1
**15**	5500	80	3160	143	605 ± 23
**16**	9500	94	4520	92.5	82 ± 3.7
**17**	21,000	125	>50,000	390	507 ± 26
**18**	164	46	4435	3250	226 ± 13
**19**	109	33	475	6760	91 ± 4.4
**20**	6	2	363	9880	85 ± 2.7
**21**	69	11	4550	4060	95 ± 3.9
**22**	164	46	1985	25,200	85 ± 2.6
**23**	109	33	282	>50,000	89 ± 3.4
**24**	95	30	294	>50,000	84 ± 1.5
**AAZ**	250	12	445	816	94 ± 3.0
**MZA**	50	14	421	8140	91 ± 6.2
**EZA**	25	8	440	3170	95 ± 2.8
**DCP**	1200	38	1220	5790	73 ± 3.0
**DZA**	50,000	9	360	742	274 ± 14
**BRZ**	45,000	3	451	739	61 ± 1.4
**BZA**	15	9	2115	410	54 ± 0.9
**TPM**	250	10	414	673	363 ± 23
**ZNS**	56	35	1820	1885	710 ± 40
**SLP**	1200	40	1715	670	493 ± 27
**IND**	31	15	4240	216	94 ± 4.6
**VLX**	54,000	43	4425	3730	831 ± 38
**CLX**	50,000	21	2513	857	669 ± 29
**SLT**	374	9	3210	496	4838 ± 257
**SAC**	18,540	5959	5280	7075	191 ± 12
**HCT**	328	290	3350	6680	545 ± 41

^a^ From references [7,8]; ^b^ from reference [17]; ^c^ this work: mean ± standard error, from three different assays.
